# Safety and preliminary efficacy of argatroban plus dual antiplatelet therapy for acute mild to moderate ischemic stroke with large artery atherosclerosis

**DOI:** 10.1002/brb3.2664

**Published:** 2022-06-08

**Authors:** Xiao‐Qiu Li, Xiao‐Wen Hou, Yu Cui, Xiao‐Fu Tian, Xin‐Hong Wang, Zhong‐He Zhou, Hui‐Sheng Chen

**Affiliations:** ^1^ Department of Neurology General Hospital of Northern Theater Command Shenyang P.R. China

**Keywords:** anticoagulant, dual antiplatelet, early neurological deterioration, ischemic stroke, large artery atherosclerosis

## Abstract

**Objective:**

Previous studies suggest the benefit of dual antiplatelet therapy (DAPT) for acute ischemic stroke with large artery atherosclerosis (LAA) etiology, but there is no study about the effect of DAPT plus anticoagulant in this population.

**Methods:**

A prospective single arm trial was performed to determine the effect of DAPT combined with argatroban on acute mild to moderate ischemic stroke patients with LAA, which was compared with historical populations. The main outcome was the proportion of early neurological deterioration (END). The secondary outcomes included scores of 0 to 1 and 0 to 2 on the modified Rankin Scale (mRS) at 90 days, and changes in National Institutes of Health Stroke Scale (NIHSS) from baseline to day 7 after admission. The safety outcomes included intracranial hemorrhage at 7 days, organ hemorrhage, and all‐cause mortality at 90 days.

**Results:**

A total of 120 patients with argatroban plus DAPT were prospectively enrolled and 529 patients with only DAPT were retrospectively collected. There was no significant difference in baseline characteristics between groups. Compared with control group, combined treatment group had lower proportion of END (4.2% vs. 10.0%, adjusted *p *= .046), more reduction in NIHSS score from the baseline to day 7 after admission (1.06 ± 2.03 vs. 0.39 ± 1.97, adjusted *p* = .003), and higher proportion of mRS (0–2) at 90 days (87.5% vs. 79.2%, adjusted *p* = .048). No intracranial hemorrhage was found between groups.

**Conclusions:**

This is the first report that short‐term argatroban combined with DAPT seems to be safe and may effectively prevent END and improve neurological prognosis for acute mild to moderate ischemic stroke patients with LAA; however, interpretation of the conclusion required caution due to nonrandomized controlled trial with medium sample size.

## Graphical abstract

Graphical Abstract:

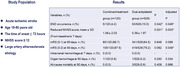

## 1 INTRODUCTION

Large artery atherosclerosis (LAA) accounts for about 25% of acute ischemic stroke (AIS). This type of stroke is apt to progress and reoccur, with result of higher disability and higher mortality (Ge et al., 2020; Ois et al., 2013). Early reperfusion therapy such as intravenous thrombolysis and endovascular treatment is the most effective method, but only a few patients can receive these treatments due to time window and technical limitations (Wardlaw et al., 2014). For the patients without receiving reperfusion treatment, antiplatelet therapy is the main treatment (Powers et al., 2018).

Recently, two clinical trials demonstrated the efficacy and safety of aspirin plus clopidogrel in patients with acute minor stroke. The Clopidogrel in High‐Risk Patients With Acute Nondisabling Cerebrovascular Events (CHANCE) trial showed that compared with aspirin monotherapy, clopidogrel plus aspirin treatment could reduce the risk of recurrent stroke in patients with transient ischemic attacks (TIA) or minor ischemic stroke (National Institutes of Health Stroke Scale, NIHSS ≤ 3) within 24 h of symptom onset (Y. Wang et al., 2013), which is further demonstrated by Platelet‐Oriented Inhibition in New TIA and Minor Ischemic Stroke (POINTS) trial (Johnston et al., 2018). Furthermore, LAA stroke was found to possibly benefit more from dual antiplatelet therapy (Kim et al., 2019; C. Wang et al., 2015; Yi et al., 2014). Even if patients were given dual antiplatelet treatment, some of them still suffered from early neurological deterioration (END), which is closely related with poor outcome. How to prevent END in AIS patients, especially LAA stroke, has become a hot issue in clinical research.

Anticoagulant therapy has been used in the acute phase of stroke, although its use is still controversial (Liang et al., 2018; M. Liu et al., 2018). Anticoagulant combined with antiplatelet therapy has been the routine antithrombotic strategy for acute coronary syndrome (Gurbel et al., 2019; Kirolos et al., 2019), which shares similar mechanism with LAA stroke. Based on these considerations, we present a proposal that short‐term combination of anticoagulant and dual antiplatelet therapy should be feasible and effective for AIS patients with LAA (Hou & Chen, 2020). However, to our best knowledge, there is no study focusing on the effect of their combination in this population.

In the present study, we aimed to investigate the efficacy and safety of the short‐term combination of argatroban and dual antiplatelet therapy (DAPT) in acute mild to moderate ischemic stroke patients with LAA.

## 2 METHODS

### 2.1 Study design

The prospective, open label, single arm study was conducted from October 2017 to January 2019, and approved by the ethics committee of General Hospital of Northern Theatre Command (former General Hospital of Shenyang Military Region, IRB: k(2017)38). Signed informed consents were obtained from the patients, or their legally authorized representative. The patients received a continuous argatroban infusion for 2–5 days and DAPT for 3 months. To determine the possible effect of the combination treatment on cerebral microbleed, some patients also received susceptibility weighted imaging (SWI) examination before and 7 days after treatment. The inclusion criteria included: (1) age 18–80 years old; (2) clear diagnosis of ischemic stroke patients with head computerized (CT) or magnetic resonance imaging (MRI) examination; (3) the time of onset ≤ 72 h; (4) NIHSS score ≤ 12; (5) with LAA etiology, according to the definition of Trial of Org 10172 in Acute Stroke Treatment (TOAST) classification (Adams et al., 1993), which was identified based on medical history and extensive workup such as intra‐ and extracranial vascular imaging (magnetic resonance angiography, carotid ultrasonography, or CT angiography), 12‐lead electrocardiography or 24 h Holter electrocardiography, and transthoracic echocardiogram to exclude other etiology; (6) signed informed consent. The exclusion criteria included: (1) patients with planned thrombolytic or endovascular therapy; (2) serious diseases such as severe infection or liver, kidney, hematopoietic system, and endocrine system; (3) the history of stroke and had serious sequelae (modified Rankin Scale, mRS > 1); (4) allergic to aspirin/clopidogrel and argatroban; (5) ischemic stroke caused by other causes, such as small vessel lesions, cardiogenic embolism, arterial dissection, vasculitis, and other cerebral infarction; (6) history of cerebral hemorrhage; (7) it is expected to use other antiplatelet agents or nonsteroidal anti‐inflammatory agents that affect platelet function; (8) within 3 months of gastrointestinal bleeding or major surgery; (9) any unqualified patients judged by researchers.

Historical control patients who met the same inclusion/exclusion criteria and only received DAPT were screened, and the following data were obtained from the electronic database (electronic medical records by hospital information system): age, gender, history of hypertension, diabetes, coronary heart disease, stroke, NIHSS score at admission and 7 days after hospitalization, onset‐to‐treatment time, TOAST classification, organ hemorrhage, intracranial hemorrhage, and so forth. The historical control patients were collected between March 2015 and January 2019 in the same hospital. In the control patients, the informed consent was waived given the retrospective nature of the analysis.

The data underlying this article will be shared upon reasonable request to the corresponding author.

### 2.2 Interventions

In the prospective single arm trial, all patients without AF history who received MRA or CTA examination within 48 h after onset were screened. Patients meeting inclusion and exclusion criteria will receive a continuous argatroban infusion for 2–5 days. Intravenous 100 μg/kg argatroban bolus is administrated over 3 to 5 min, followed by argatroban infusion of 1.0 μg/kg per minute. These infusion rates of argatroban are adjusted to a target activated partial thromboplastin time (APTT) of 1.75 × baseline (± 10%) (Sugg et al., 2006). For the first day, clopidogrel with loading dose 300 mg and aspirin 100 mg were given, and followed by clopidogrel 75 mg and aspirin 100 mg each day. The combined treatment was finished during hospitalization.

### 2.3 Data collection

All patients will be assessed at admission, 7 days after hospitalization, and 90 days poststroke. At admission, patients underwent a complete evaluation including age, gender, smoking status, alcohol consumption, history of hypertension, diabetes, coronary heart disease, and stroke; onset‐to‐treatment time, NIHSS score at admission and at 7 days after hospitalization, TOAST classification, organ hemorrhage, intracranial hemorrhage, and death were collected; at 90 days poststroke, mRS was collected by face‐to‐face or telephone interview.

### 2.4 Efficacy and safety outcomes

The primary efficacy outcome was the proportion of END, which was defined as 1 or more increase in NIHSS at 7 days compared with the baseline, because the patients with mild or moderate neurological deficit were enrolled in the present study. The secondary efficacy outcomes included the reduction in NIHSS score from the baseline to day 7 after admission, mRS (0–1), and mRS (0–2) at 90 days. The safety outcomes were intracranial hemorrhage at 7 days, organ hemorrhage, and all‐cause mortality at 90 days. Intracranial hemorrhage was defined as acute extravasation of blood into the brain parenchyma or subarachnoid space with associated neurologic symptoms 5, which was confirmed by brain CT or MRI.

### 2.5 Standard protocol approvals, registrations, and patient consents

All participants or their legal proxies provided written informed consent, and the ethics committee of General Hospital of Northern Theater Command approved the study. The trial was in accordance with the Declaration of Helsinki and registered (NCT02609256).

### 2.6 Statistical analysis

For continuous variables that were normally distributed, data were presented as mean and standard deviation. When variables were not normally distributed, data were presented as median and interquartile range. Count variables were described with *n* (%). For the comparison of continuous variables, t‐test or Wilcoxon rank sum test was used. Chi‐square test was used to compare count variables. Multivariate logistic regression model was used to adjust for baseline imbalances, and all the variables in baseline were used as a covariate in adjusted analysis. SPSS 20.0 was used to perform the analysis.

## 3 RESULTS

**FIGURE 1:**
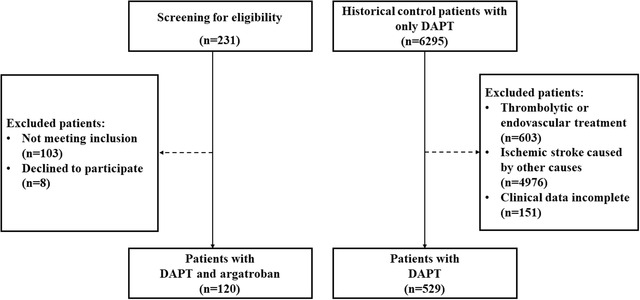
Flow diagram of the participants selection. *Abbreviation*: DAPT, duel antiplatelet therapy.

Between October 2017 and January 2019, 120 eligible patients were enrolled and received argatroban plus DAPT after excluding 111 patients (103 who did not meet inclusion criteria and 8 who declined to participate) (Figure 1). After consecutively screening 1879 historical patients with acute mild to moderate stroke from March 2015 to January 2019 in our stroke center, 529 control patients who met the same inclusion/exclusion criteria and only received DAPT were finally recruited to the current study as control group (Figure 1).

The baseline characteristics of patients between two groups are shown in Table 1. No significant difference was found between two groups. Of 120 patients with combined treatment, 32 patients received SWI examination before and 7 days after treatment. The duration of argatroban in the combined treatment group was 2.0 (2.0–3.0) days.

**TABLE 1:** Characteristics of the participants

Variables	Combined treatment group (*n* = 120)	Dual antiplatelet group (*n* = 529)	*p*
Age, years, mean ± SD	60.9 ± 8.5	61.7 ± 9.8	.377
Males, *n* (%)	88/120 (73.3)	380/529 (71.8)	.741
Current smoker, *n* (%) Current drinker, *n* (%)	65/120 (54.2)	261/529 (49.3)	.340
	59/120 (49.2)	229/529 (43.3)	.242
Admission NIHSS, median (IQR)	4.0 (2.0‐6.0)	3.0 (1.0‐6.0)	.149
OTT (h), mean ± SD	30.5 ± 18.9	32.4 ± 22.1	.325
Hypertension, *n* (%)	80/120 (66.7)	353/529 (66.7)	.989
Diabetes, *n* (%)	39/120 (32.5)	170/529 (32.1)	.939
Coronary heart disease, *n* (%)	15/120 (12.5)	64/529 (12.1)	.903
Stroke history, *n* (%)	37/120 (30.8)	170/529 (32.1)	.782
Anterior circulation infarction, *n* (%)	84/120 (70.0)	366/529 (69.2)	.458
ICA, *n* (%)	47/120 (39.2)	219/ 529 (41.4)	
MCA, *n* (%)	35/120 (29.2)	91/529 (17.2)	
ACA, *n* (%)	2/120 (1.6)	56/529 (10.6)	
Posterior circulation infarction, *n* (%)	36/120 (30)	121/529 (22.9)	.282
BA, *n* (%)	6/120 (5.0)	21/529 (4.0)	
VA, *n* (%)	18/120 (15.0)	90/529 (17.0)	
PCA, *n* (%)	12/120 (10.0)	10/529 (1.9)	

Abbreviations: ACA, anterior cerebral artery; BA, basilar artery; ICA, internal carotid artery; IQR, interquartile range; MCA, middle cerebral artery; NIHSS: National Institutes of Health Stroke Scale; OTT, onset‐to‐treatment time; PCA, posterior cerebral artery; SD, standard deviation; VA, Vertebral artery.

As shown in Table 2, more END occurred in DAPT group, compared with combination treatment group (4.2% in combination treatment group, 10.0% in DAPT group, adjusted *p* = .046). More reduction in NIHSS score from the baseline to day 7 after admission was found in combination treatment group (1.06 ± 2.03 in combination treatment group vs. 0.39 ± 1.97 in DAPT group, adjusted *p* = .003). More proportion of mRS (0–2) at 90 days was found in combination treatment group (87.5% in combination treatment group vs. 79.2% in DAPT group, adjusted *p* = .048). Similar proportion of mRS (0–1) at 90 days was shown between two groups. No intracranial hemorrhage was found between two groups. No significant difference of organ hemorrhage or death at 90 days was found between two groups (Table 2). In addition, the combination treatment had no effect on cerebral microbleed in patients receiving SWI examination. No significant difference in the baseline characteristics was not found between patients with SWI examination or not (Table 3).

**TABLE 2:** The efficacy and safety of combined therapy versus dual antiplatelet

Variables, *n* (%)	Combined treatment group (*n* = 120)	Dual antiplatelet group (*n* = 529)	*p*	*Adjusted p*
END occurrence, *n* (%)	5/120 (4.2)	53/529 (10.0)	.042*	.046*
Reduced NIHSS score, mean ± SD (From baseline to 7 days)	1.06 ± 2.03	0.39 ± 1.97	.001*	.003*
mRS (0‐1) at 90 days, *n* (%)	80/120 (66.7)	341/529 (64.5)	.648	0.088
mRS (0‐2) at 90 days, *n* (%)	105/120 (87.5)	419/529 (79.2)	.062	.048*
Intracranial hemorrhage at 7 days, *n* (%)	0 (0.0)	0 (0.0)	–	–
Organ hemorrhage at 90 days, *n* (%)	1/120 (0.8)	1/529 (0.2)	.250	.334
All‐cause mortality at 90 days, *n* (%)	0 (0.0)	3/529 (0.6)	.408	.996

Abbreviations: mRS, modified Rankin Scale, NIHSS, National Institutes of Health Stroke Scale.

* *p* < .05.

**TABLE 3:** The characteristics of the patients with vs without SWI examination

Variables	Patients with SWI (*n* = 32)	Patients without SWI (*n* = 88)	*p*
Age, years, mean ± SD	59.4 ± 8.2	61.1 ± 8.6	.438
Males, *n* (%)	25/32 (78.1)	64/88 (72.5)	.550
Current smoker, *n* (%) Current drinker, *n* (%)	22/32 (72.2)	45/88 (51.0)	.086
	20/32 (62.5)	41/88 (46.6)	.123
Admission NIHSS, median (IQR)	3.0 (2.0–6.0)	4.0 (2.0–6.0)	.450
Onset‐to‐treatment time, mean ± SD	29.0 ± 18.0	30.7 ± 19.1	.716
Hypertension, *n* (%)	23/32 (71.9)	58/88 (65.9)	.537
Diabetes, *n* (%)	12/32 (37.5)	28/88 (31.8)	.559
Coronary heart disease, *n* (%)	5/32 (15.6)	10/88 (11.4)	.533
Stroke history, *n* (%)	14/32 (43.8)	25/88 (28.4)	.113
Anterior circulation infarction, *n* (%)	20/32 (62.5)	63/88 (71.6)	.340
Posterior circulation infarction, *n* (%)	11/32 (34.4)	23/88 (26.1)	.376

Abbreviations: NIHSS, National Institutes of Health Stroke Scale; SWI, susceptibility weighted imaging.

## 4 DISCUSSION

Anticoagulant combined with dual antiplatelet is very aggressive treatment for acute ischemic stroke, and there is lack of the related studies. For the first time, the current prospective study investigated the safety and possible efficacy of the combination strategy for AIS patients with LAA. The results showed that the short‐term antithrombotic combination is safe and may prevent END and improve neurological prognosis in this population.

Stroke has been the first leading cause of death in China with high disability and recurrence rates (Tu et al., 2021), while END is closely associated with death and disability (P. Liu et al., 2020). In the present study, the END rate of the patients in the combined treatment group was significantly lower than that in the dual antiplatelet group, which suggests that argatroban combined with dual antiplatelet may effectively prevent END. In addition, we found that the combined treatment produced a significant reduction in NIHSS from the baseline to day 7 after admission, and more proportion in patients with mRS 0–1 and mRS 0–2 on day 90 after onset, respectively. The results suggest that the combined treatment may improve early and long‐term neurological prognosis in this population. No intracranial hemorrhage including cerebral microbleed was found in the participants in our study, which may be attributed to the short‐term combination of antithrombosis. Collectively, the findings suggest that the short‐term argatroban combined with dual antiplatelet therapy was safe and may improve neurological prognosis for acute mild to moderate stroke patients with LAA.

As we know, antiplatelet therapy is the main strategy for AIS with LAA (Ois et al., 2013; Sandercock et al., 2008). Although anticoagulant therapy has been used for more than 50 years, its use is still controversial. Warfarin Aspirin Symptomatic Intracranial Disease (WASID) trial demonstrated that warfarin provided no benefit over aspirin due to increased rate of adverse events (Chimowitz et al., 2005). Several randomized trials showed that anticoagulant therapy could reduce stroke recurrence, pulmonary embolism, and deep vein thrombosis, but its efficacy might be offset by a high incidence of symptomatic intracranial hemorrhage (Sandercock et al., 2008). Thus, the precise antithrombotic strategy based on different etiology of stroke should be the future direction.

Several studies have found that patients with LAA stroke might benefit more from dual antiplatelet therapy (Kim et al., 2019; C. Wang et al., 2015; Yi et al., 2014). Two retrospective studies found that argatroban combined with antiplatelet improved the NIHSS at discharge without significant symptomatic cerebral hemorrhage (Chen et al., 2018) and prevented the incidence of progressing stroke (Nishi et al., 2016). Our recent study suggested that short‐term combination of argatroban with dual antiplatelet therapy appeared safe in acute minor posterior circulation ischemic stroke (Zhou et al., 2020). In theory, under the premise of controlling the risk of bleeding, the greater the intensity of antithrombotic therapy, the less the recurrence and aggravation of stroke. Taken together, we argue that the short‐term combination of argatroban and dual antiplatelet therapy may be the suitable antithrombotic strategy for acute mild to moderate stroke patients with LAA, which would be helpful to reduce high occurrence rates of disability and death in the stroke patients (Tu et al., 2021).

The present study has several characteristics, which are different from previous studies. First, this is the first prospective study to investigate the effect of anticoagulant combined with dual antiplatelet in specific target population (acute mild–moderate stroke with LAA). Second, argatroban combined with dual antiplatelet was used. Argatroban was chosen based on two considerations: (1) its direct inhibition of thrombin in blood clots, rapid action, short duration of action, low bleeding tendency, and no immunogenicity (Guy et al., 2018; Jing et al., 2016); (2) clinical monitoring of anticoagulation levels: the dose of argatroban would be adjusted to a target APTT of 1.75 × baseline in the present study. Third, patients with minor to moderate stroke (NIHSS score ≤ 12 points) within 72 h after onset were enrolled in the present study. The design aimed to cover more patients who potentially benefited from the dual antiplatelet plus argatroban. Finally, a short‐term combination was used in the present study. We argue that the combination treatment administered for 2–5 days is enough to cover the time window of high risk of END (Toni et al., 1997), and the short‐term combination will decrease the risk of bleeding events.

Our study has several limitations to address. First, it is a single‐center study, which may have some bias. Second, this is a single arm study, although historic control was assigned and adjusted by multivariate logistic regression model. Third, the sample size is relatively small. Fourth, END was defined as 1 or more increase in NIHSS at 7 days compared with the baseline, given that the patients with mild or moderate neurological deficit were enrolled in the present study. However, the small change in total NIHSS might reflect inadequate reliability rather than true END in the current study. Finally, only 32 of 120 patients performed SWI, so the effect of combined antithrombotic treatment on cerebral microbleed was not fully demonstrated.

## 5 CONCLUSIONS

The prospective study provided the first report that short‐term argatroban combined with dual antiplatelet therapy was safe and may prevent early neurological deterioration and improve neurological prognosis for acute mild to moderate ischemic stroke patients with LAA; however, interpretation of the conclusion required caution due to non‐randomized controlled trial with medium sample size. Multicenter, randomized controlled and large sample size studies are warranted to investigate the treatment regimen in this population.

## CONFLICT OF INTEREST

The authors have no conflicts of interest to disclose.

## AUTHOR CONTRIBUTIONS

First draft and revision of the manuscript: Xiao‐Qiu Li. Analysis and interpretation of data and first draft of the manuscript: Xiaowen Hou. Check of analysis and interpretation of data: Yu Cui. Collection of data: Xin‐Hong Wang and Zhong‐He Zhou. The study design and critical revision: Hui‐Sheng Chen.

### PEER REVIEW

The peer review history for this article is available at https://publons.com/publon/10.1002/brb3.2664

## Data Availability

The data that support the findings of the study are available from the corresponding author on reasonable request.
